# Selections

**Published:** 1898-12

**Authors:** 


### SELECTIONS.

### STATE EXAMINATION OF MILK FOR TUBERCULOSIS.

Dr. Florince O. Donohue, of Syracuse, reviewed what had been done in this direction, more especially by New York State, which, though the pioneer worker in this field, was to-day by no means in advance of other States. He said that of all the animals that are susceptible to tubercular infection, the cow is most to be dreaded by man, as cow's milk is consumed by infants and sick persons whose digestion is so impaired that they fall a ready prey to the tubercle bacillus. Tubercle bacilli can always be found in milk from a cow having a tuberculous udder, and they are sometimes detected in milk from animals whose udders are free from the disease. A sufficient impetus to this much-needed work of inspecting the herds and destroying the tuberculous animals can be found in the statistics of the State Board of Health, which show that over 12,000 deaths in the State are caused annually by tuberculosis. Like other reforms, the first step was beset with difficulties, but the "tuberculosis act," passed in 1892, gave the board authority to inspect all cattle in the State and destroy those found to be tuberculous. The intereference with dairy interests and the pecuniary loss sustained by the owners of condemned cattle soon started an opposition, so that the work was brought to a halt. The law is still operative in New York State, and the commissioners are drawing their salaries, but the lack of adequate appropriation has kept the work practically at a standstill for the past two years. As soon as the board became convinced of the diagnostic value and the harmlessness of the tuberculin-test, it was made use of freely, and it has been abundantly proved a great advance on mere dependence on physical signs. In the course of a year and a half 22,000 head of cattle were inspected and 700 destroyed. Most of this work was done in the Hudson River district. In most cases it was possible to trace the disease from herd to herd, and no breed of cattle appeared to be exempt from the ravages of tuberculosis. Tuberculosis in the cow is, as a rule, a chronic disease, and, consequently, animals afflicted with it may yield a fair milk for a considerable time. The fact that milk is practically the sole article of diet of artificially-fed infants, in conjunction with the distribution of the tuberculous lesions in childhood, has aroused the suspicion that tuberculous milk is the source of infection in such children.

Dr. Hiram A. Pooler, of New York, said that he had observed a difference between the milk from cows properly fed and that from those kept on brewers' feed, and he has also noted that tuberculosis is more prevalent in districts where cattle are given such unwholesome food. As a result of his arguments in this direction before the Legislature a law was enacted in 1883 prohibiting the use of such unwholesome food for cattle.

Dr. H. O. Marcy, of Boston, spoke of the interesting experiments that had been recently conducted with a view to eliminating pathogenic bacteria from milk by a process known as refrigeration. The milk is agitated while it is freezing, and this process of ice-crystallization is kept up for several hours. The product is almost free from objectionable bacteria, and, as it contains only about 7 per cent. of water, it is a good deal thicker than cream. It is not at all improbable that in the near future it will be possible to so perfect this process, and carry it out on a commercial scale, that solidified milk will become an ordinary article of commerce, just like butter. From what is known of refrigerated milk, as at present prepared, its keeping qualities are excellent.

Dr. A. T. Van Vranken thought an important link in the evidence adduced regarding the danger to the human race from tuberculous cattle is missing, unless it has been proved that there is a distinct relation between bovine and human tuberculosis in the same localities.

Dr. S. A. Knopf, of New York, said he has become a convert to the view that the chief channel of infection in the human subject is the digestive tract.

Dr. Donohue, in closing the discussion, said that while bovine tuberculosis has been found to be exceedingly prevalent in the eastern part of the State, it is comparatively rare in the western part. Reasoning from analogy, with the evidence furnished by experiments repeatedly made on animals, the missing link referred to by one speaker has been supplied.—*Society Proceedings, New York State Medical Association, in the Philadelphia Medical Journal*, October 29, 1898.

Long, President of the Board of Agriculture, believes that the veterinary profession can do a great deal toward forwarding the movement for diminishing tuberculosis apart from any step that it might be necessary for the Government to take. The Government should provide tuberculin at a comparatively small cost and pay a fee for its employment, in order to secure properly qualified veterinary surgeons to carry it out. A proper application of such measures would eradicate tuberculosis in cattle, and indirectly to a great extent in man.—*Ibid.*

WISE gives some general hygienic directions for the avoidance of tuberculosis, viz., careful selection of a properly situated dwelling upon which plenty of sunlight falls, which is elevated, but not windswept, and, particularly, one that is not damp. The furnishings should be light, and, when feasible, washable. Servants should not be allowed to "dust," but should wipe furniture after dampening it, and the carpets should be dampened and then cleaned with a sweeper. Breathing should be through the nose; the clothing should be loose, moderately light, but warm. Sufficient exercise should be taken, and a proper diet should be prescribed. The expectoration should be sterilized. Milk and meats should be chosen with great care, excluding, if possible, tuberculous infection. The final point is of importance. It is pointed out that numerous animals used as pets, as well as several domestic animals, are susceptible to tuberculosis, and it is particularly insisted that, among these, canaries and others are frequent subjects of tuberculosis.—*Ibid.*

VARIATION IN THE STRENGTH OF CRUDE DRUGS..—The following variation in the alkaloidal strength of crude drugs bought in the open market by a large firm of manufacturing chemists and analyzed by them is here given: Calabar bean: physostigmine found to vary from 0.16 to 0.3 per cent. Cantharides: cantharidine found to vary from 1 to 4 per cent. Cinchona, calisaya: total alkaloids found to vary from 3.5 to 10 per cent. Colchicum root: colchine found to vary from 0.3 to 0.6 per cent. Hyoscyamus: hyoscyamine found to vary from 0.7 to 13 per cent. Male fern: filicic acid found to vary from 0.22 to 0.65 per cent. Nux vomica: brucine and strychnine found to vary from 2.5 to 3.5 per cent.—*Therapeutic Notes*, P. D. & Co.

VITALITY OF TYPHOID BACILLUS.—From recent investigations, it has been ascertained that the bacillus of typhoid fever will live and retain all of the characteristic virulence for a period of three months when kept in a dry condition.

